# Supplementary figures and images for: Transcriptome-based analysis of the hormone regulation mechanism of gender differentiation in Juglans mandshurica Maxim
Source: PeerJ. 2021 Nov 9;9:e12328. doi: 10.7717/peerj.12328 (PMC8588858; doi:10.7717/peerj.12328)

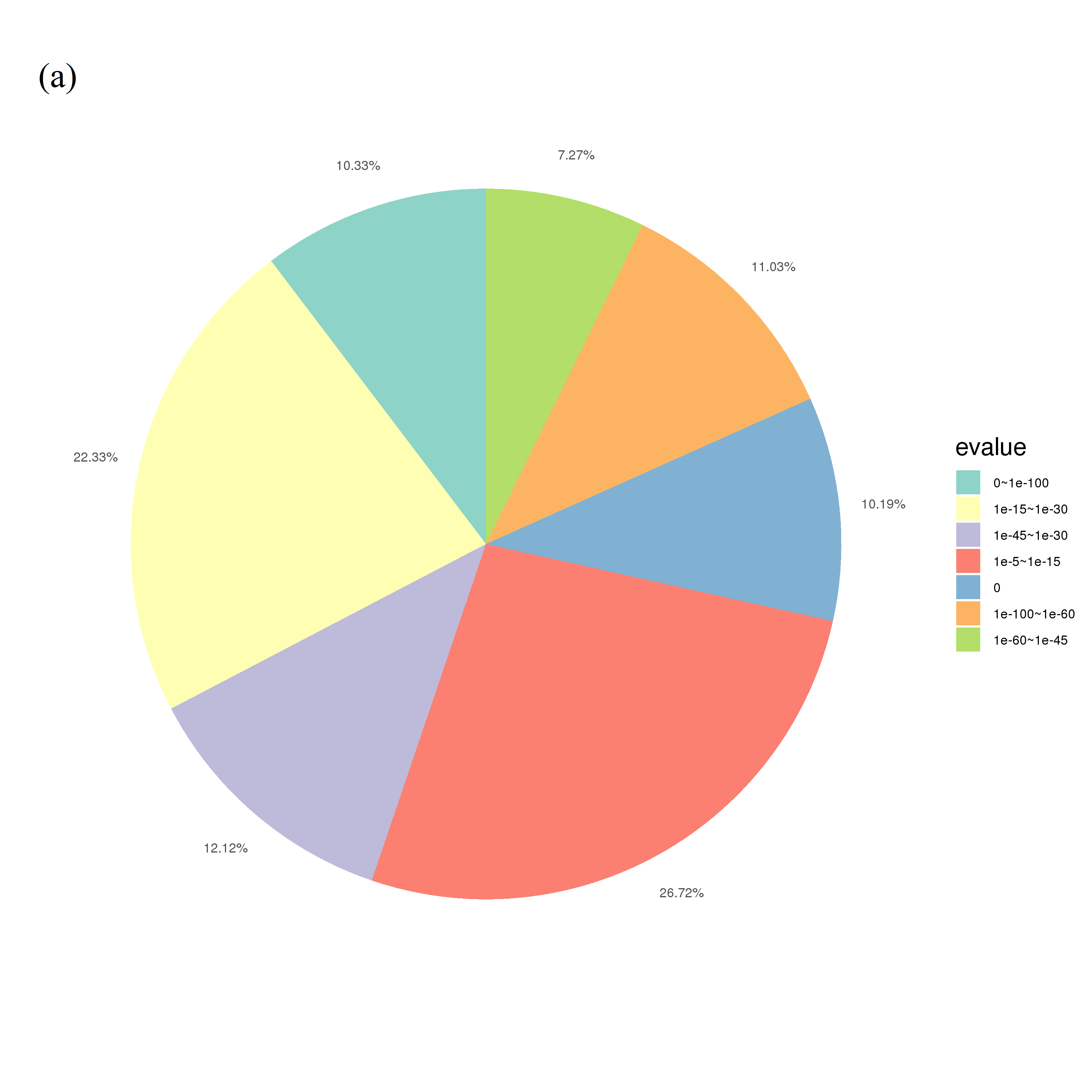

Supplement: Supplemental Information 1 [file peerj-09-12328-s001.png]

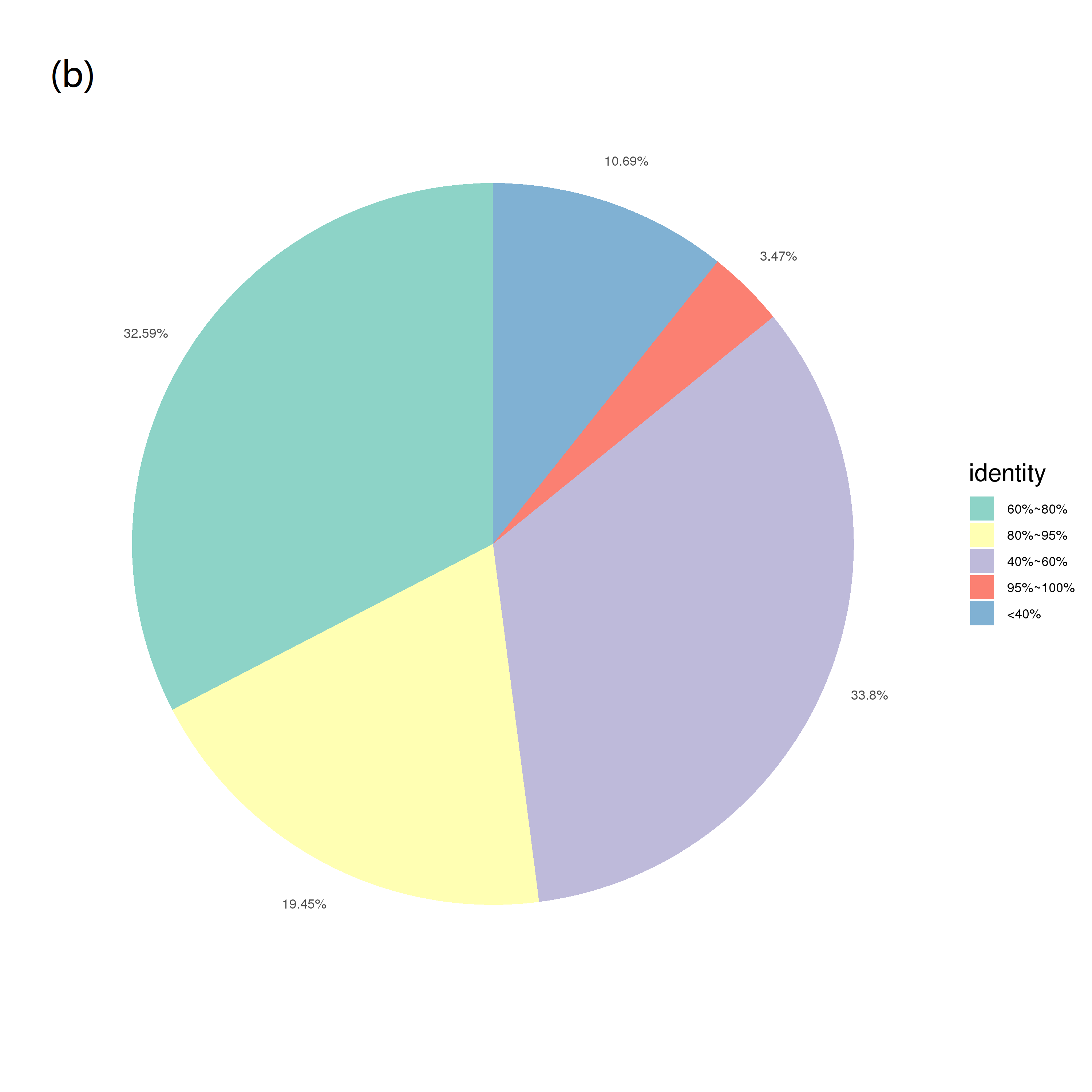

Supplement: Supplemental Information 2 [file peerj-09-12328-s002.png]

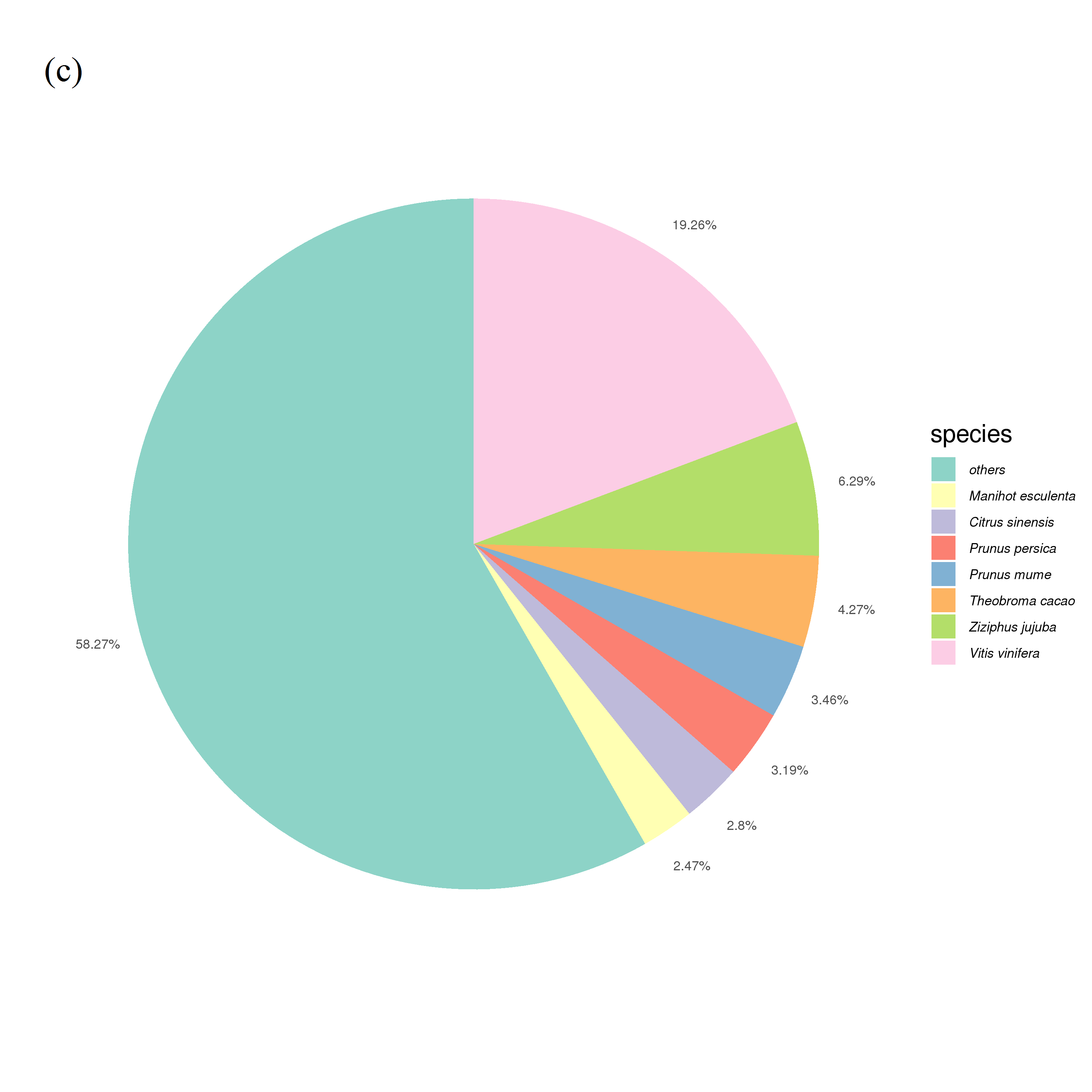

Supplement: Supplemental Information 3 [file peerj-09-12328-s003.png]

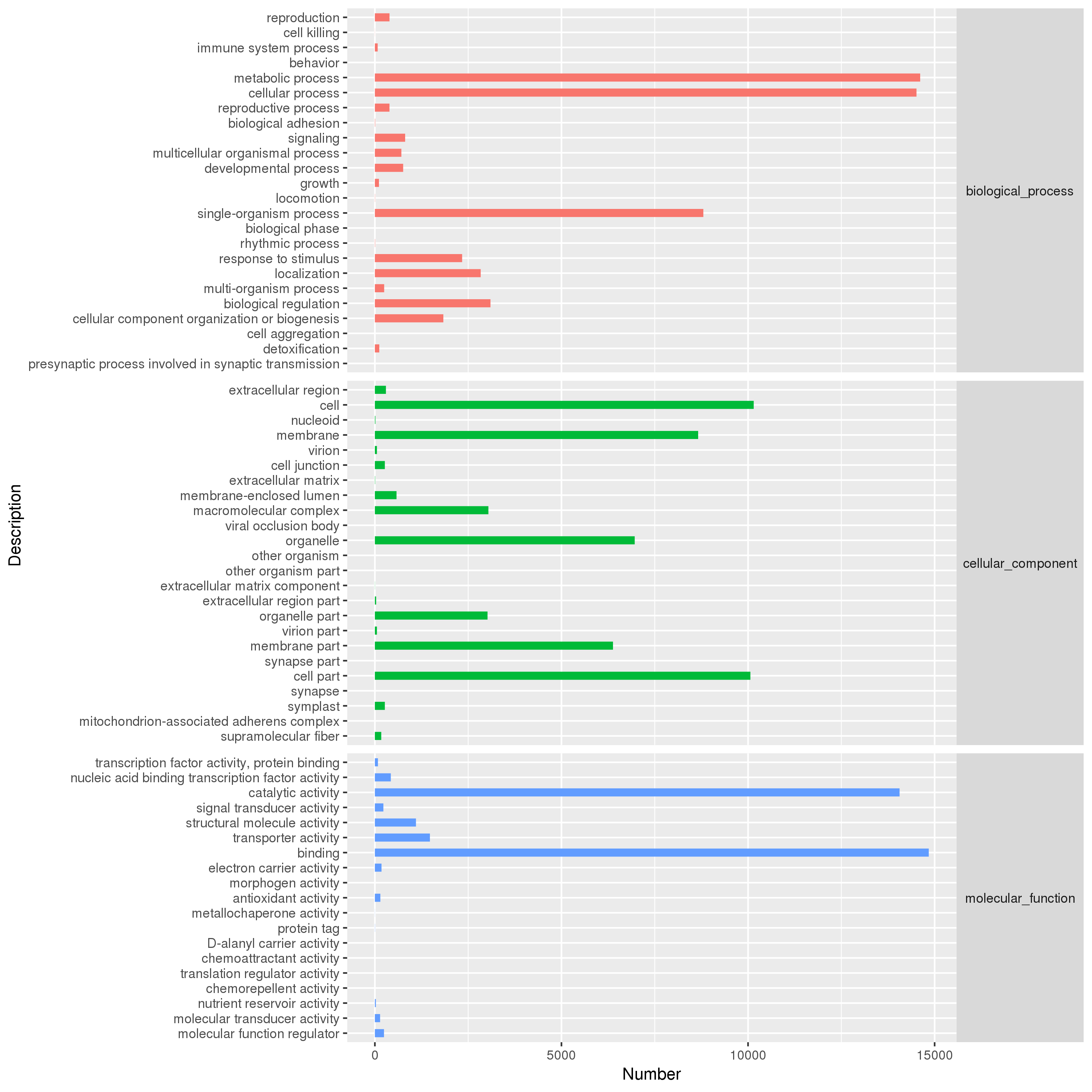

Supplement: Supplemental Information 4 [file peerj-09-12328-s004.png]

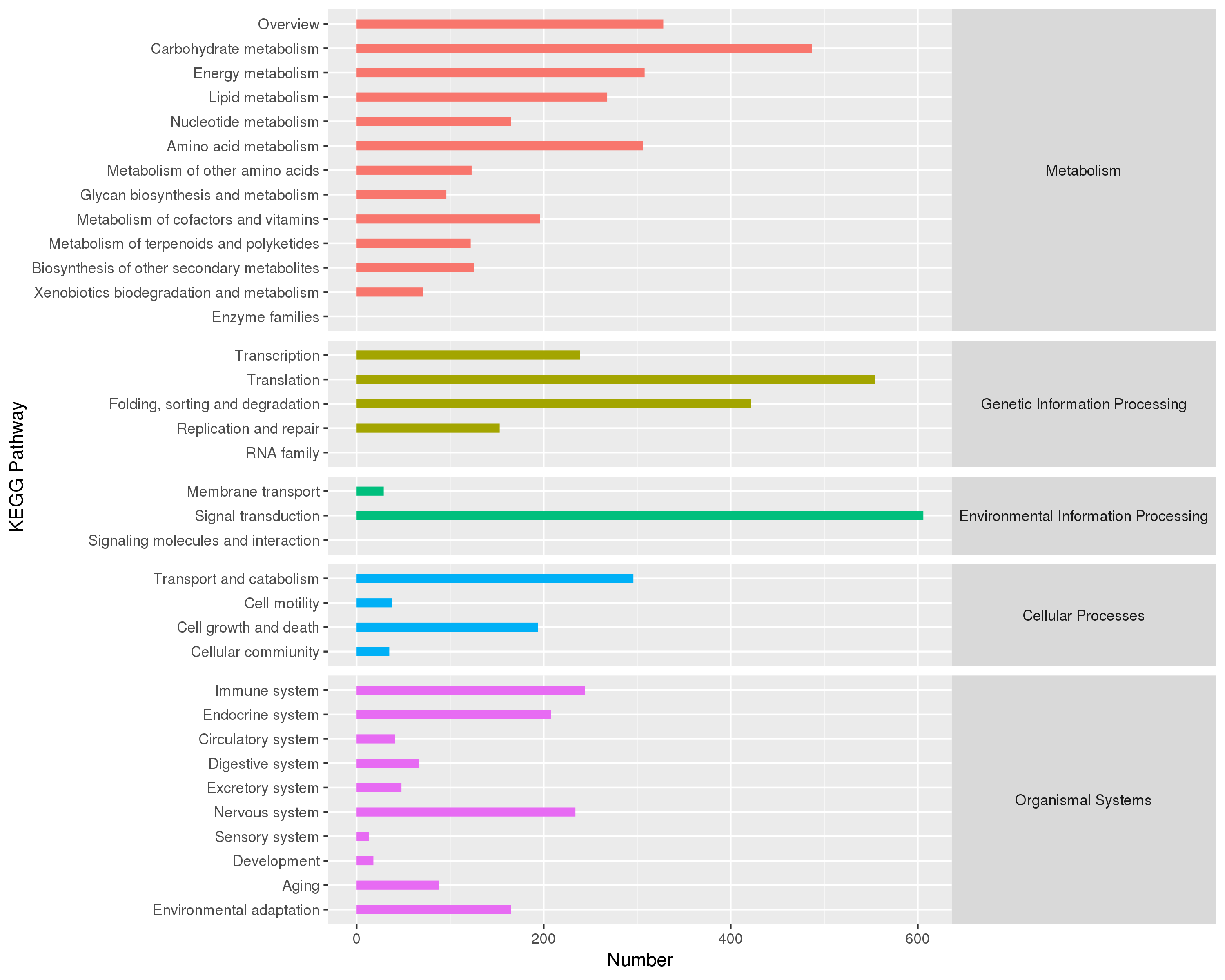

Supplement: Supplemental Information 5 [file peerj-09-12328-s005.png]
