# Supplementary material for: Transcriptome-based analysis of the hormone regulation mechanism of gender differentiation in Juglans mandshurica Maxim
Source: PeerJ. 2021 Nov 9;9:e12328. doi: 10.7717/peerj.12328 (PMC8588858; doi:10.7717/peerj.12328)
Supplement: Supplemental Information 6 [file peerj-09-12328-s006.docx]

**Table S1.** Statistics of clean reads for transcriptome in *Juglans mandshurica.*

| Sample | Clean Reads No. | Clean Data(bp) | Clean Reads% | Clean Data% |
| --- | --- | --- | --- | --- |
| A1  A2  A3  A4  A5  A6  B1  B2  B3  B4  B5  B6 | 46469762  44337254  43894726  45290776  43720762  40977488  41042740  44672674  42772328  41614774  46025550  42795778 | 6991929010  6676989782  6610627884  6821069232  6583578838  6171705464  6181197576  6723473430  6439756908  6265843792  6932567412  6442665752 | 99.49  99.41  99.42  99.41  98.94  99.07  99.06  98.80  99.14  99.02  99.11  99.08 | 99.13  99.14  99.16  99.15  98.67  98.82  98.80  98.48  98.85  98.74  98.87  98.79 |
